# Radioresistance in rectal cancer: can nanoparticles turn the tide?

**DOI:** 10.1186/s12943-025-02232-x

**Published:** 2025-01-30

**Authors:** Diogo Coelho, Diogo Estêvão, Maria José Oliveira, Bruno Sarmento

**Affiliations:** 1 i3S - Instituto de Investigação e Inovação em Saúde, Universidade Do Porto, Rua Alfredo Allen 208, Porto, 4200‑135 Portugal; 2 INEB - Instituto de Engenharia Biomédica, Universidade Do Porto, Rua Alfredo Allen 208, Porto, 4200‑135 Portugal; 3 IUCS – Instituto Universitário de Ciências da Saúde, CESPU, Rua Central de Gandra 1317, Gandra, 4585-116 Portugal; 4 Laboratory of Experimental Cancer Research, Department of Human Structure and Repair, Cancer Research Institute, Ghent University, Ghent, Belgium; 5 ICBAS – Instituto de Ciências Biomédicas Abel Salazar, Universidade do Porto, Rua Jorge Viterbo Ferreira, Porto, 4200-319 Portugal

**Keywords:** Rectal cancer, Treatment, Chemoradiotherapy, Radioresistance, Nanomedicines

## Abstract

Rectal cancer accounts for over 35% of the worldwide colorectal cancer burden representing a distinctive subset of cancers from those arising in the colon. Colorectal cancers exhibit a continuum of traits that differ with their location in the large intestine. Due to anatomical and molecular differences, rectal cancer is treated differently from colon cancer, with neoadjuvant chemoradiotherapy playing a pivotal role in the control of the locally advanced disease. However, radioresistance remains a major obstacle often correlated with poor prognosis. Multifunctional nanomedicines offer a promising approach to improve radiotherapy response rates, as well as to increase the intratumoral concentration of chemotherapeutic agents, such as 5-Fluorouracil. Here, we revise the main molecular differences between rectal and colon tumors, exploring the complex orchestration beyond rectal cancer radioresistance and the most promising nanomedicines reported in the literature to improve neoadjuvant therapy response rates.

**Graphical Abstract:**

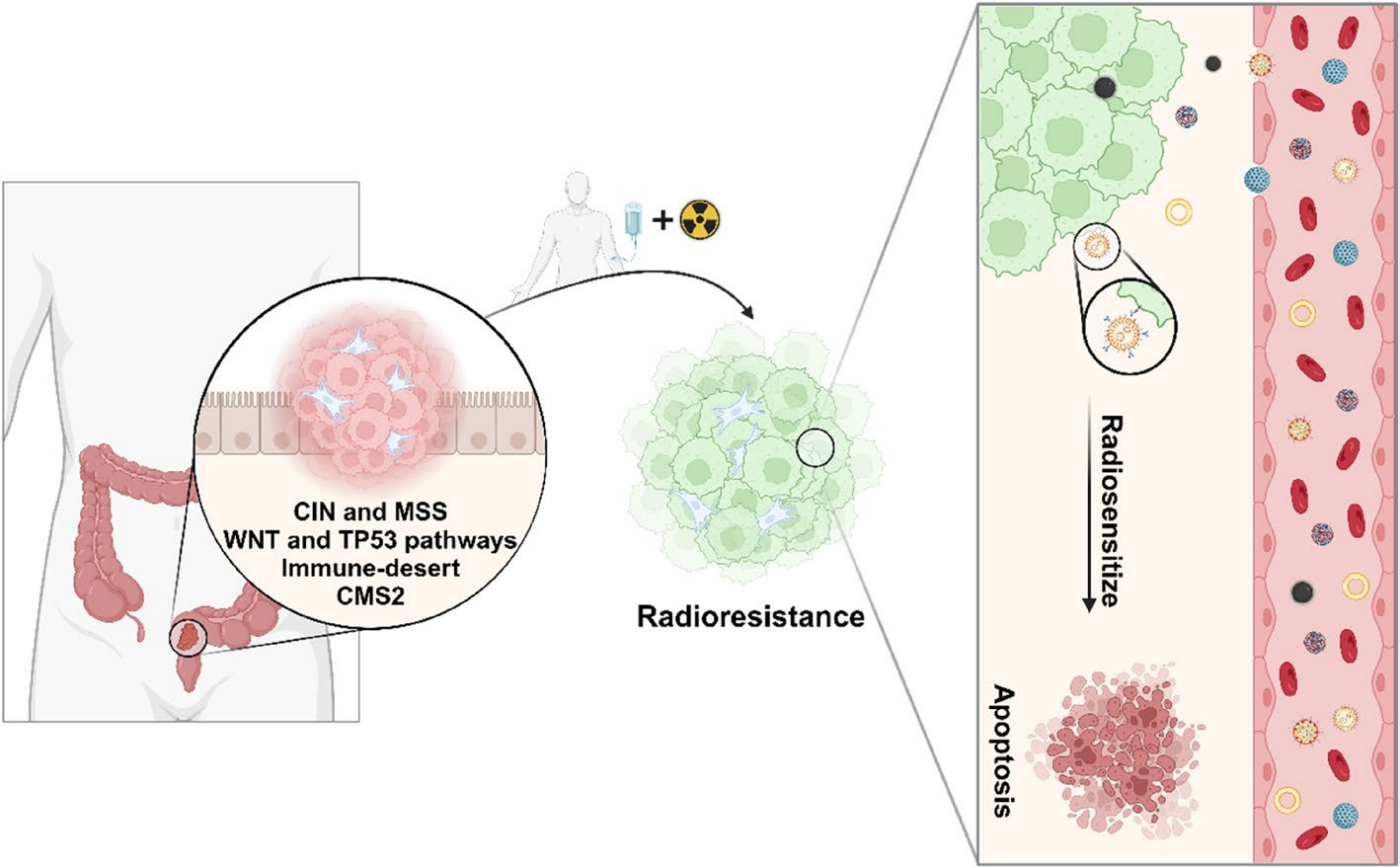

## Background

Cancer is a heterogeneous disease characterized by a multiplicity of cellular and acellular components, driving gene mutations responsible for the well-described hallmarks proposed by Hanahan and Weinberg that are crucial for tumor cells to shift from a normal to a neoplastic state [1, 2]. Colorectal cancer (CRC) is a global public health challenge, responsible for almost 2 million new cancer cases and 10% of all cancer-related deaths worldwide [3]. Moreover, CRC burden is projected to rise by 63% in the next two decades [4]. Within CRC, rectal cancer (RC) accounts for more than 35% of all new CRC cases and deaths, thus contributing to the high incidence and mortality rates of gastrointestinal malignancies [3]. Alarmingly, incidence among young adults, who are not currently included in routine screening guidelines, have nearly doubled over the last decades, positioning CRC as the leading cause of cancer death among young adult men [5]. Notably, RC is a major driver of this concerning trend, and by the end of the current decade, an increase of 124% in RC patients, is expected, under the age of 35 years [6]. Furthermore, nearly a quarter of all new RC cases are expected to be diagnosed in individuals younger than 50 years of age, often at an advanced stage [6]. In addition, males more frequently present left-sided colon and rectal tumors, while females exhibit increased risk of right-sided colon tumors [7, 8]. High estrogen levels seem to protect young women from developing CRC, however other mechanisms cannot be excluded [8]. Lifestyle habits have played a significant role in the high incidence of CRC among younger patients. Lack of physical activity, along with high consumption of red and processed meats, as well as iron and heme-rich substances, has been linked to an increased risk of disease development and progression [9].

Treatment of RC frequently relies on neoadjuvant radiotherapy as around 50% of the patients present advanced disease at diagnosis, requiring pre-operative ionizing radiation to shrink the tumor and improve loco-regional control [10]. However, despite the most recent advances in the field of ionizing radiation, as high as 40% of RC patients do not respond or only partly respond to the therapeutical scheme [11]. However, in recent years, multifunctional nanomedicines have emerged as promising tools to enhance the therapeutic efficacy of radiotherapy in RC [12, 13]. Indeed, nanosystems offer the potential for targeted drug delivery, addressing a major challenge in cancer treatment: specific drug accumulation within tumors. Nanoparticles (NPs) benefit from reduced lymphatic drainage and increased vascular permeability, the so-called enhanced permeability and retention (EPR) effect [14]. Besides, NPs can be surface modified with ligands, such as antibodies or peptides, to improve their target ability to tumor cells [15]. In this review, we not only explore the anatomical and molecular differences behind rectal and colon carcinogenesis, outlining their clinical implications, but we also delve into the complex landscape of radioresistance in RC, discussing the potential offered by nanomedicines to enhance preoperative therapy outcomes in the context of RC.

## Colon and rectal cancer: two entities or the same?

**Fig. 1:**
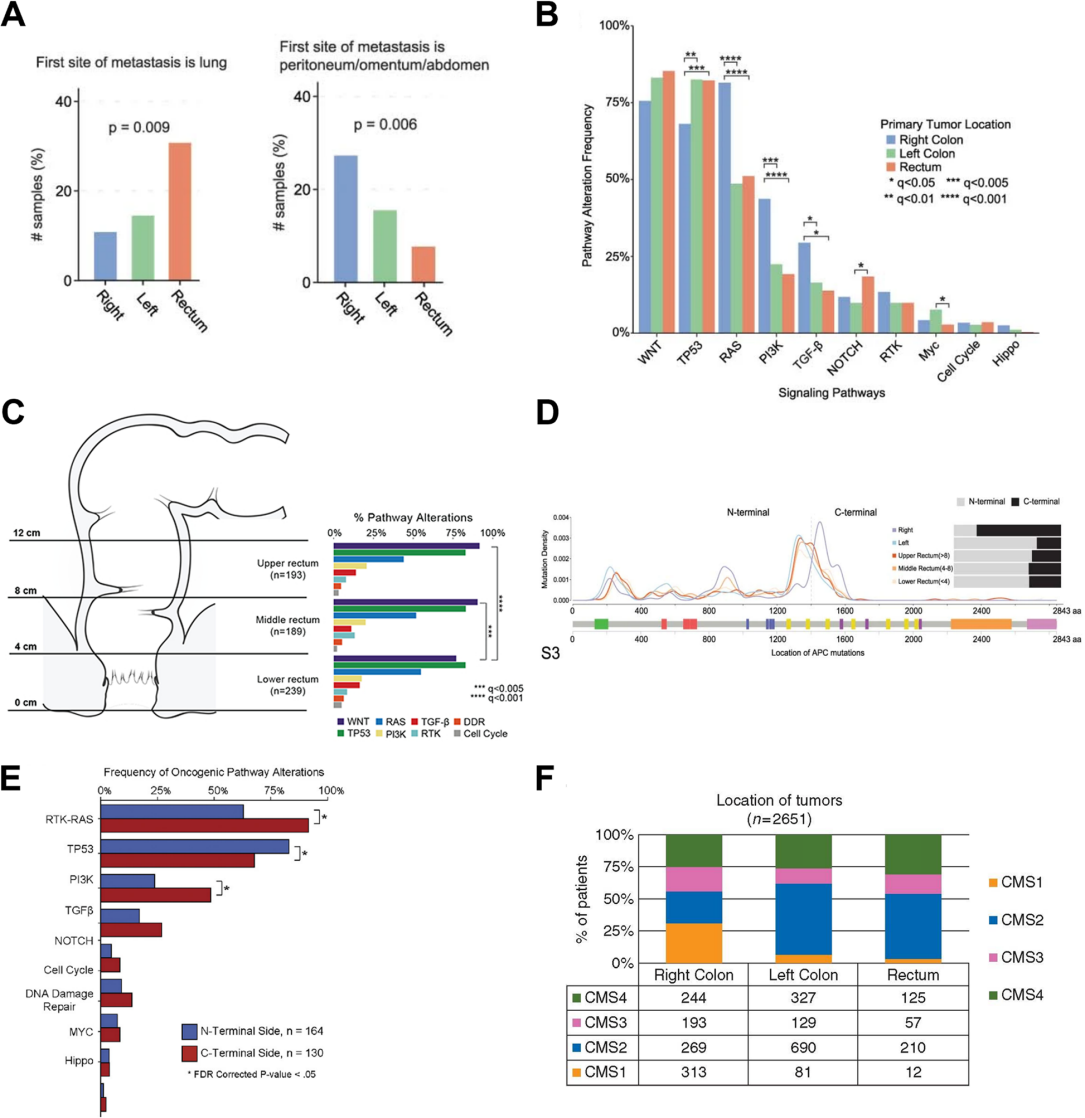
Complex genetic landscape between colon and rectal tumors. **A** Metastasis patterns differences between colon and rectal tumors. **B** Signaling pathways alterations according to tumor location, **C** and over the rectum. **D** Genomic location of *APC* mutations in tumors arising from the right colon to rectum (upper, middle and lower). *q < 0.05, **q < 0.01, ***q < 0.005, and ****q < 0.001. Reproduced (Adapted) with permission from Chatila et al. [27]. Copyright 2022, Springer Nature. **E** Correlation between signaling alterations and *APC* mutation side. *p < 0.05. Reproduced (Adapted) with permission from Mondaca, Walch et al. [28]. Copyright 2020, American Gastroenterological Association Institute **F** CMS distribution throughout CRC. Reproduced (Adapted) under terms of Creative Commons Attribution-NonCommercial 4.0 International (CC BY-NC 4.0) license from Fontana et al. [29]. Copyright 2019, The authors, Published by Oxford University Press on behalf of the European Society for Medical Oncology. APC, Adenomatous polyposis coli; CMS, consensus molecular subtype; CRC, colorectal cancer, PI3K, phosphoinositide 3-kinase; TP53, tumor protein p53

Historically, cancers arising along the large intestine have been grouped altogether as CRC. Although often discussed as a single disease, emerging evidence suggests that colon cancer (CC) and RC exhibit cellular and molecular differences with relevant clinical impact. While anatomically continuous, the colon and rectum hold anatomical, physiological and embryological differences [16]. These discrepancies directly influence how CRC develops and spreads through the body. While the colon extends throughout the large intestine, from the cecum to the sigmoid colon, the rectum represents the end part of the large bowel up to 15 cm from the anal verge, being divided into three regions, the lower (0–5 cm from the anal verge), middle (5–10 cm) and the upper rectum (10–15 cm) [10]. Venous drainage is also a key difference, with a direct influence on the metastasis patterns of both cancers. Unlike the colon and upper rectum, which are venously drained by the portal hepatic system, the middle and lower rectum drain directly into the inferior vena cava through the internal iliac vein, bypassing the liver and reaching the lungs directly, which contributes for RC higher risk of lung metastasis compared to CC [17–19]. In fact, RC patients generally present a higher risk of extra-abdominal metastasis, while CC more frequently spreads within the abdomen, namely to the liver and peritoneum (Fig. 1A) [20, 21]. A study covering 1675 patients with metastatic colorectal cancer, concluded that RC patients presented more frequently brain metastases than those with CC (5.0% versus 2.6%, respectively) [21]. Concomitantly, a nationwide Swedish registry-based study revealed that besides a higher risk of nervous system metastasis, rectal tumors are 1.38 more likely to spread to the bone [22]. Nevertheless, it is still under debate whether these metastasis patterns are due solely to anatomical differences, or if molecular traits between both cancer types play a role. Moreover, even though CC is the most common, accounting for approximately two-thirds of all CRC cases, the risk of developing RC is anatomically four times greater, due to the smaller surface area of the rectal mucosa to the colon [23]. Rectal physiology also plays a crucial role in the higher risk of cancer observed in this area. The colon is mainly responsible for water and nutrients absorption, as well as feces formation, whereas the rectum temporarily stores them until defecation [24]. This rectum slower intestinal flow leads to a prolonged exposure of the rectal mucosa to digestion end products and to potential carcinogens present in the feces, which may contribute to its higher cancer risk. Additionally, the embryological origin of the large bowel is heterogeneous. While the proximal colon stems from the midgut, the distal colon and rectum arise from the hindgut [25, 26]. This heterogeneity may contribute to the observed similarities in molecular pathways involved in rectal carcinogenesis and distal CC, which differ from those observed in proximal CC [27].

Colorectal carcinogenesis is a multistage process that involves the accumulation of genetic and epigenetic alterations. Those mechanisms are essential to silence and activate tumor suppressor genes and oncogenes, respectively, fostering stem cells, located in the crypts of Lieberkühn, or mature somatic cells transition to cancer stem cells, which are major drivers for the initiation and progression of the colorectal carcinogenesis [30, 31]. CRCs develop through three distinct carcinogenic sequences: adenoma-carcinoma sequence, serrated pathway and inflammatory pathway, closely related to three molecular pathways: chromosomal instability (CIN) pathway, microsatellite instability (MSI) pathway, and CpG island methylator phenotype (CIMP) pathway, which describe the molecular events that drive cancer progression [32, 33]. The adenoma-carcinoma sequence, proposed in 1990 by Fearon and Vogelstein, is a multi-hit genetic model that outlines the natural history of most CRCs [31]. This sequence delineates the orderly progression of events and mutations in specific genes, triggering a tightly regulated proliferative state shift of the mucosa to a hyperproliferative one, culminating in the malignant transformation of the colorectal mucosa [31]. The CIN pathway explains the molecular alterations in 65–70% of the sporadic CRCs and is characterized by structural and numerical chromosomal abnormalities, such aneuploidy and loss of heterozygosity in tumor suppressor genes [34]. Additionally, several studies have shown that the majority of RC exhibit not only these traits, but also microsatellite stability (MSS) and CIMP absence, underlining the importance of the CIN pathway in RC development [35–37]. Moreover, it is observed a gradual change in such features from the rectum to the proximal colon, where cancers often exhibit MSI and CIMP^+^ [38]. This was shown by Yamauchi and his colleagues, evidencing a progressive increase in the prevalence of cancers with MSI and CIMP^+^ from the rectum (< 2.3%) to the ascending colon (36–40%) [39]. Microsatellite sequences are short nucleotide tandem repeats in DNA, considered hotspots for mutations. Hypermethylation of the promoter region or mutations in mismatch repair (MMR) genes, such *MutL Homolog 1* (*MLH1*) or *MutS Homolog 2* (*MSH2*) cause instability in those regions due to the inability to repair mutations that may arise in these sequences, allowing cell division while keeping those mutations. In contrast, the CIMP pathway is characterized by frequent methylation of cytosine and guanine dinucleotide clusters, the so-called CpG islands, often located in the promoter regions of tumor suppressor genes, such as *MLH1*, resulting in mismatch repair deficiency (dMMR) [32]. Interestingly, a recent study comprising 287 CRC patients who underwent MSI analysis also revealed a progressive increase in MMR-related gene mutations from the rectum to the ascending colon, as 22.3% of right-sided colon tumors exhibited a dMMR, while only 7.1% and 0.7% of distal colon and rectal tumors did, respectively [40]. These findings correlate with the previous ones and explain why MSI and CIMP^+^ CRCs are more frequently observed in the proximal colon. Moreover, although CRC is considered a type of cancer with low mutational burden, those arising in the colon, particularly in its proximal segment, exhibit a higher mutation rate, being considered hypermutated, compared to RC [41]. This aligns with the higher prevalence of MSI-high (MSI-H) tumors in this region, characterized by a greater number of mutations, as a result of a dMMR which impairs cell ability to intrinsically repair mutations. Strikingly, a genomic analysis of 224 CRC tumors pinpointed a considerable difference in the mutational landscape between hypermutated and non-hypermutated tumors. Notably, key genes for CRC progression like the Tumor protein p53 (*TP53*) (60% versus 20%) and the Adenomatous Polyposis Coli (*APC*) (81% versus 51%) were significantly highly mutated in non-hypermutated tumors [42].

One of the most striking examples is the p53 protein, referred to as the guardian of the genome, encoded by the *TP53* gene, the most mutated gene in cancer, and is critical in cell division and survival regulation [43]. *TP53* mutations are a hallmark in CRCs that arise via the CIN pathway and are usually found only in colorectal adenocarcinomas, highlighting the critical role this gene plays in the progression of a late adenoma to the carcinoma stage [31]. In 2014, Russo et al. concluded in a study involving 222 patients with metastatic CRC that *TP53* mutations were significantly more common in patients with rectal adenocarcinomas (29.7%) than CC (17.7%) [44]. Moreover, the same authors found a correlation between *TP53* mutations and young-onset RC [44]. Additionally, RC more frequently exhibit alterations in the WNT/β-catenin signaling pathway than CC [27, 45]. Dysregulation of the WNT-β-catenin pathway is an early hallmark in colorectal carcinogenesis. The multi-protein complex APC-GSK3β-Axin acts as a gatekeeper for this pathway by binding to the transcriptional coactivator β-catenin and promoting its degradation in the cytoplasm, maintaining it at basal levels. Thus, mutations in genes related to this complex, such as *APC*, or the *Catenin Beta 1* (*CTNNB1*) gene, lead to its constitutive activation, accumulation of cytoplasmic β-catenin with subsequent translocation into the nucleus, promoting gene expression and leading to aberrant proliferation and differentiation [46]. Kapiteijn et al. demonstrated that RC express more nuclear β-catenin than CC [45]. Moreover, *APC* mutations were reported to be significantly more frequent in CRC arising in the rectum than those from the proximal colon [40]. Recently, Chatila and colleagues pointed out the same trend as somatic alterations in the WNT signaling pathway being more frequent from the ascending colon to the rectum **(**Fig. 1B) [27]. The same study also showed that among 621 RC patients, the prevalence of alterations along the WNT pathway significantly decreased from the upper to the lower rectum, emphasizing the importance of tumor location and molecular pathways driving rectal carcinogenesis may vary across the organ (Fig. 1C) [27]. Interestingly, it was also noted that most *APC* mutations in the distal colon and rectum occur in the N-terminus of the gene, while proximal CC exhibited a higher frequency of C-terminus mutations (Fig. 1D) [27]. Also, it was previously established that the mutation location in the *APC* gene influences the cellular processes affected. In 2020, a study conducted by Mondaca, Walch and colleagues assessed the impact of *APC* mutation site on the survival of metastatic CRC patients with MSS and found that C-terminal mutations were correlated with a shorter overall survival and progression-free survival [28]. The authors propose that the worst prognosis observed in CRC patients harboring *APC* C-terminal mutations is associated with the co-occurrence of alterations in genes involved in mitogenic pathways such as the Mitogen-Activated Protein Kinase (MAPK) and the Phosphoinositide 3-Kinase (PI3K) signaling pathways (Fig. 1E) [28]. Indeed, the literature widely supports the hypothesis that rectal and distal colon tumors are more TP53 and APC signaling dependent, while those arising in the proximal colon are driven by mutations in genes involved in the ERK/MAPK, PI3K and TGF-β pathways [28, 47–49].

Another distinct trait observed between colon and rectal adenocarcinomas is the predominance of mutations in *v-Raf murine sarcoma viral oncogene homolog B1* (*BRAF*). This gene encodes a serine/threonine protein kinase (B-Raf) that plays a critical role in the ERK/MAPK signaling pathway regulation, known to control cell proliferation, differentiation and survival. Several studies have shown that *BRAF* mutations are rare during RC carcinogenesis, being more regularly found in CC, particularly in the ascending ones, located on the right-side [39, 44]. Furthermore, *BRAF* alterations are described as an early event in the CIMP pathway, closely related to the serrated pathway [50]. Interestingly, cancers arising from serrated adenomas, characterized by their distinctive stellate pattern of crypt infolding, are predominantly found in the proximal colon, showing the prominent role that the CIMP pathway plays in the carcinogenesis of proximal CC, in discrepancy with the rectum, where serrated adenomas are rare [51].

The molecular heterogeneity observed in CRCs translates into differences in the surrounding tumor microenvironment (TME). Although most CRCs are considered low immunogenic, exhibiting low T-cell infiltration, tumors harboring MSI-H and a dMMR, often found in the ascending colon, usually present high immune infiltration as a result of their high tumor burden, being associated with better prognosis [52]. This was recently confirmed by Mezheyeuski et al. in a cohort study, which evaluated the immune landscape in CRC by multiplex immunofluorescence and stated that colon tumors, mainly those on the right side where mostly MSI-H with increased infiltration of CD4^+^ and CD8^+^ T cells compared to RC [53]. These findings explain why RC is predominantly classified, according to the Consensus Molecular Subtype (CMS) classification, into the CMS2 or canonical subtype, characterized by their low immune infiltration (immune-desert), MSS and CIMP-low, frequently harboring *WNT* and *TP53* mutations (Fig. 1F) [29, 54, 55].

**Fig. 2:**
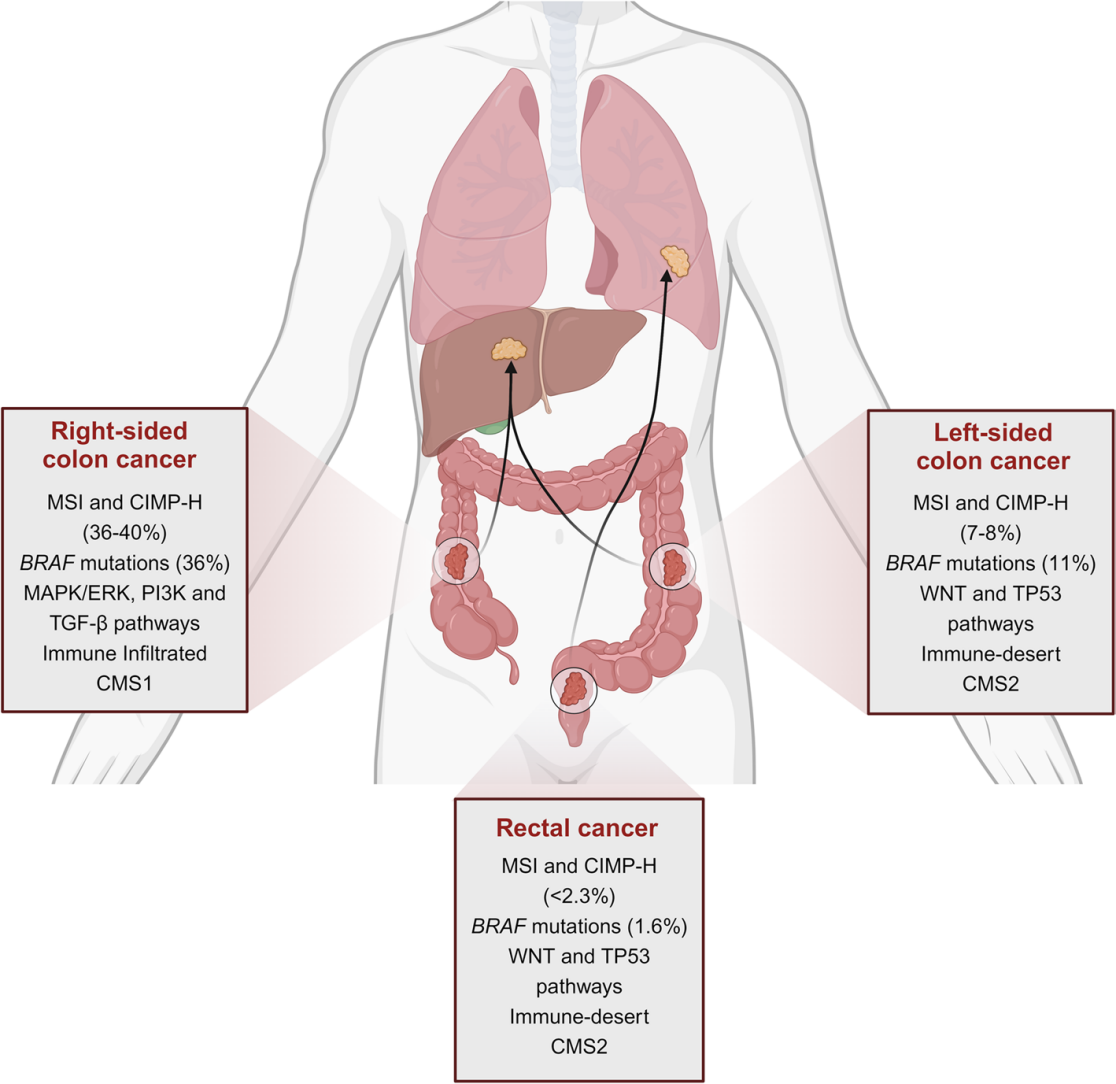
Schematic representation of the molecular differences between rectal cancer and colon cancer

Overall, although often considered the same, cellular and molecular characteristics of colon and rectal tumors emphasize that they should be considered as two distinct entities. CRC exhibit a unique genetic profile according to the location across the large bowel. RC is strongly associated with a CIN-dependent progression compared to right-sided CC, often characterized by a MSI and CIMP-H phenotype, leading to increased tumor immunogenicity (Fig. 2). Therefore, RC is managed as a distinct clinical entity from CC.

## The complex landscape of rectal cancer therapy: a challenge for disease management

The molecular heterogeneity between cancers arising from the colon and the rectum translates into divergent therapeutic approaches applied in the non-metastatic panorama of both diseases. Early diagnosis is strongly associated with improved prognosis, empowering the adoption of a curative approach through surgical removal of the tumor. Total mesorectal excision stands as the cornerstone of curative intent for RC, enabling remarkable advances in the survival of these patients [56]. Extramural vascular invasion and positive circumferential resection margins, namely tumor proximity to the mesorectal fascia, are the two strongest predictors for local recurrence in RC [57, 58]. Therefore, high-resolution imaging techniques, particularly magnetic resonance imaging, play a pivotal role in RC management, being described, according to the last European Society for Medical Oncology (ESMO) guidelines for RC treatment, as the most reliable technique to locoregional stage RC and assess local recurrence and distant metastases risk [10]. Still, due to the anatomical location of the rectum within the posterior pelvis, close to the bladder, prostate, vagina and uterus, locoregional recurrence risk in RC is comparatively higher than in CC [59]. Moreover, RC patients quality of life is negatively affected by the increased risk of post-surgical comorbidities, such anastomotic leak, permanent colostomy and sexual dysfunction due to rectum proximity to the sphincter muscle [60]. Consequently, surgical approaches to RC are complex and challenging, always requiring a balance between oncological outcomes and preserving the functionality of the surrounding organs.

**Fig. 3:**
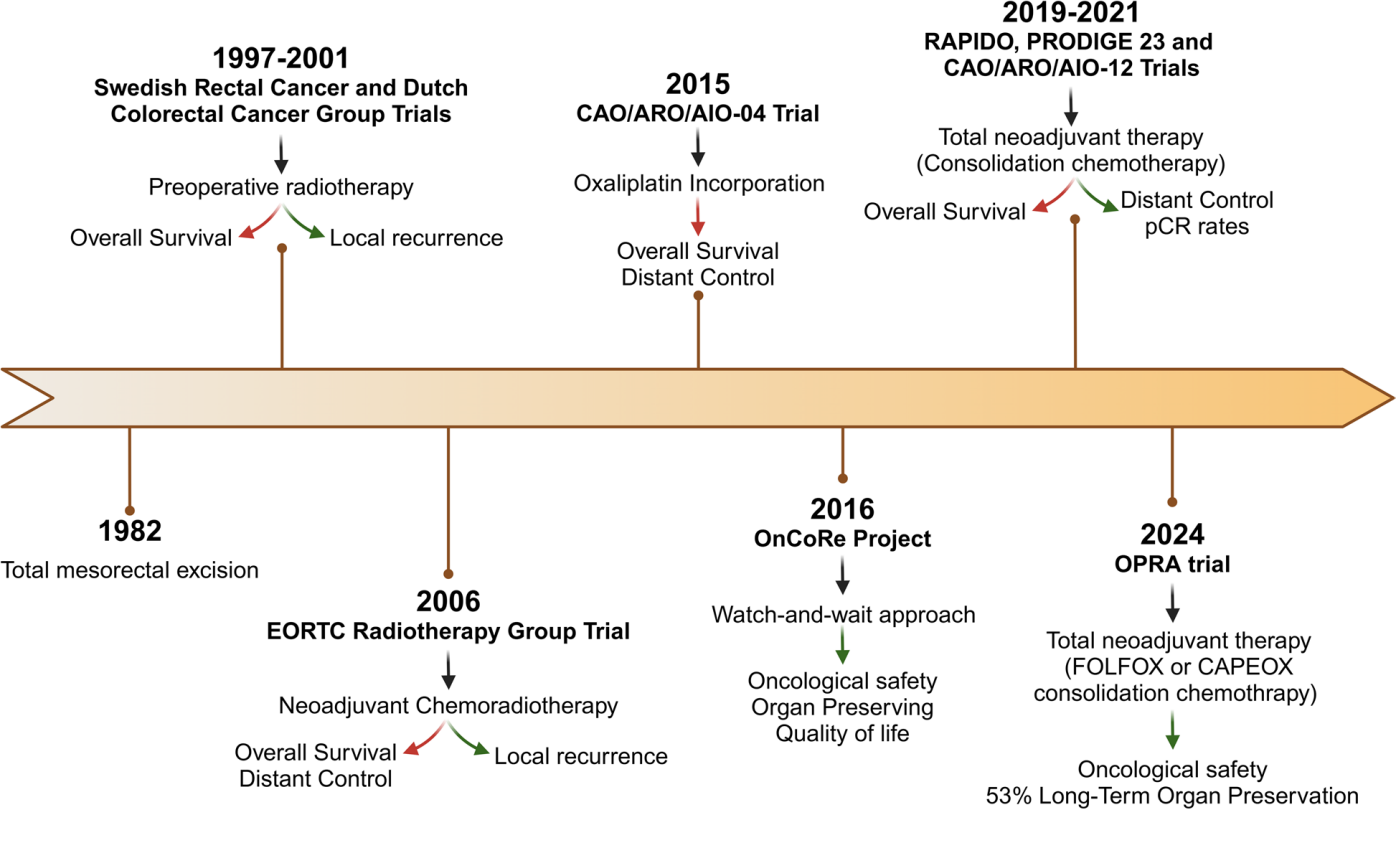
Landmark advances in rectal cancer treatment over time

Despite the significant benefits of total mesorectal excision, achieving optimal local and distant disease control remains a challenge. Seeking to improve local recurrence rates, several trials aimed to evaluate the impact of radiotherapy in a preoperative setting on local disease control compared to surgery alone. The Swedish Rectal Cancer Trial and the Dutch Colorectal Cancer Group concluded that preoperative radiotherapy followed by surgery improved local recurrence rates compared to surgery alone, and that patients undergoing only total mesorectal excision were 3.42 times more likely to experience local recurrence [61, 62]. However, no significant long-term differences in the overall survival were witnessed among those patients [63, 64]. Subsequently, attempting to increase the patients survival rates, it was found that the combination of fluorouracil-based chemotherapy to radiotherapy, so-called chemoradiotherapy, improved local control in locally advanced rectal cancer (LARC) patients, however, without any benefits for distant control and overall survival [65]. Furthermore, the multicenter German CAO/ARO/AIO-94 phase III trial concluded chemoradiotherapy benefits were superior when used preoperatively over postoperative, resulting in higher patient compliance, better local control and fewer acute and long-term toxic effects [66]. Additionally, neoadjuvant chemoradiotherapy (nCRT) supports tumor downsizing, as evidenced by a shift towards earlier TNM stages, giving the opportunity for sphincter-sparing approaches [66]. Nonetheless, despite reduced local failure associated with nCRT, achieving a good distant control of the disease and improving the overall survival among LARC patients remains challenging. Adjuvant FOLFOX4 (oxaliplatin plus infusion 5-Fluorouracil (5-FU)/leucovorin) regimens significantly improved the overall survival of stage III CC [67]. As such, the German CAO/ARO/AIO-04 study also assessed whether incorporating oxaliplatin in both nCRT and adjuvant chemotherapy could also reduce distant metastasis rates and increase LARC patients survival [68]. Remarkably, compared to several studies, such as the STAR-01, ACCORD-12 and NSABP R-04 trials, which demonstrated no improvement in disease-free survival but increased grade 3 and 4 side effects with oxaliplatin addition, the CAO/ARO/AIO-04 study reported a 3-year disease-free survival benefit in the oxaliplatin-treated group (75.9% vs. 71.2%) [68–71]. Compliance with nCRT in the oxaliplatin-treated group appears to be a critical factor contributing for these oncological outcomes disparities, as, contrarily to other trials where nCRT adherence varied between groups, adherence in the CAO/ARO/AIO-04 study was similar [72]. Nevertheless, overall survival and distant metastases remained similar between groups [68]. More promising were the outcomes observed in the RAPIDO and PRODIGE 23 trials. Both studies investigated the impact of total neoadjuvant therapy (TNT), a combination of neoadjuvant systemic 5-FU/oxaliplatin-based chemotherapy and chemoradiotherapy. While no improvement was seen in the overall survival at 3 years, in both studies, distant metastasis was a less frequent event in the group of patients treated with TNT (RAPIDO: 20% vs. 26.8%; PRODIGE 23: 17% vs. 25%) [73, 74]. Importantly, the TNT-treated group exhibited significantly higher rates of pathological complete response (pCR) (RAPIDO: 28% vs. 14%; PRODIGE 23: 28% vs. 12%), defined by the absence of malignant cells in the resected tissue, which paved the way for the adoption of an organ-preserving strategy avoiding surgery, commonly known as watch-and-wait approach [73, 74]. Besides, the CAO/ARO/AIO-12 trial noted that consolidation chemotherapy following nCRT resulted in significantly higher rates of pCR among LARC patients (25% vs. 17%), however, with no impact in long-term oncological outcomes, such as 3-year disease-free survival, locoregional recurrence or distant metastases [75, 76]. Consequently, the National Comprehensive Cancer Network (NCCN) guidelines recommend TNT for these patients [77]. The ability to improve distant control and increase the rate of patients reaching a pCR marks an important advancement in RC management, particularly in an organ-sparing approach. Looking forward, it has been observed that RC patients who exhibit a clinical complete response after neoadjuvant therapy can undergo a watchful waiting approach without compromising oncological outcomes. The OnCoRe project reported that over 60% of patients who underwent the watch-and-wait approach were able to avoid surgery while maintaining oncological safety [78]. Remarkably, even in cases of local regrowth, observed in 34% of the patients submitted to a watchful waiting approach, salvage treatment by resection was possible in 88% of cases. Furthermore, colostomies were less frequent without compromising the overall survival of those patients, resulting in a significant improvement in their quality of life [78]. Moreover, the phase II OPRA trial explored the benefits of TNT for an organ-sparing approach in stage II and III RC patients. Strikingly, nCRT followed by FOLFOX or CAPEOX consolidation chemotherapy resulted in sustained organ preservation in 53% of the cases, compared to 41% with induction chemotherapy. Importantly, disease-free survival of TNT-treated patients who underwent a watch-and-wait approach was similar to that of patients treated with nCRT followed by TME and adjuvant chemotherapy [79, 80]. Recently, the multicenter PROSPECT trial demonstrated that, compared to the gold standard nCRT, LARC patients treated with neoadjuvant FOLFOX chemotherapy achieved similar 5-year disease-free survival (80.8% vs. 78.6%), 5-year overall survival (89.5% vs. 90.2%), and pCR rates (21.9% vs. 24.3%) [81]. Surprisingly, locoregional control, often correlated with tumor irradiation, was identical between groups. These findings suggest the potential of neoadjuvant FOLFOX chemotherapy for non-advanced RC or LARC patients candidates for organ-sparing surgery, with selective pelvic irradiation for non-responders or those unable to complete the FOLFOX therapeutic scheme [81]. Nevertheless, despite recent advances in the metastatic control of RC and 5-year net survival for RC patients with metachronous metastatic disease, the 5-year overall survival rate for RC patients has barely changed over the past two decades, remaining poor, at nearly 60% [82, 83]. Figure 3 provides a summarized overview of the RC treatment evolution.

Targeted therapies and immunotherapy could offer a way to improve the survival of those patients. However, due to the molecular traits frequently found in RC, such as the low mutational rate of the *BRAF* proto-oncogene and low dependence of rectal carcinogenesis on the epidermal growth factor receptor (EGFR) signaling pathway, the effectiveness of anti-EGFR and BRAF targeted drugs is limited. Besides, while immune checkpoint inhibitors have improved the prognosis of MSI-H CRC, their effectiveness in RC is limited mainly due to the low frequency of MSI-H tumors [84]. Recently, a phase II study revealed that the addition of pembrolizumab, a programmed cell death-1 (PD-1) inhibitor, to chemoradiotherapy did not increase the pCR rate and grade 3 or 4 adverse events were more common compared to the control group [85]. Nevertheless, another study showed that among 12 LARC patients harboring dMMR, the neoadjuvant use of dostarlimab, another PD-1 inhibitor, as monotherapy increased the clinical complete response rate to 100% after six months of therapy, preventing those patients from undergoing chemoradiotherapy and surgery, with no compromise in the oncological outcomes after twelve months of follow-up [86]. Overall, despite all the advancements witnessed in RC management, the response to chemoradiotherapy varies greatly between patients. While up to one third of the patients respond well to nCRT, 20–40% of the tumors show minimal regression after ionizing radiation and the remaining patients develop a moderate response [11]. Therefore, deciphering the mechanisms underlying radioresistance and identifying predictive markers of response is therefore vital for the treatment success of RC.

### Unmasking radioresistance in rectal cancer

Radiotherapy stands as the main therapeutic modality in cancer treatment, being used in approximately 50–60% of all cancer treatments and accounting for up to 40% of cancer cures [87, 88]. Indeed, the basic principle of radiotherapy is based on the difference in DNA repair efficiency between cancer cells and normal cells. While ionizing radiation induces DNA damage that healthy cells can repair more effectively, cancer cells, on the other way, due to their impaired cellular mechanisms of DNA damage control, accumulate mutations and defects, being eventually targeted to apoptosis. This knowledge allows the development of fractionated schemes currently used in RC treatment. More specifically, the short-course preoperative radiotherapy (SCPRT) (25 Gy total dose at 5 Gy/fraction for one week) and the long-course preoperative radiotherapy (LCPRT) (45–50 Gy total dose divided by 25–28 fractions) are the gold standard schedules of preoperative radiotherapy in RC management [10]. Importantly, radiation fractionated schemes, allow hypoxic tumor cells to gain access to oxygen due to increased cell death, leading to the sensitivity of formerly hypoxic cells to irradiation [89, 90]. Nevertheless, it is unclear which scheme offers the best clinical outcomes. The RAPIDO trial revealed that while reducing distant metastasis and increasing pCR rates, the SCPRT-treated group experienced slightly higher locoregional failure rates than nCRT based on a LCPRT scheme (8.3% vs. 6.0%) [73]. Additionally, a Polish phase 3 trial assessed the effectiveness of SCPRT followed by FOLFOX4 chemotherapy compared to LCPRT with concurrent oxaliplatin and 5-FU/leucovorin [91]. SCPRT group exhibited less acute toxicity and improved 3-year overall survival, yet not sustained, as long-term revealed similar 8-year overall survival (49%) [91, 92]. Concerningly, most RC patients do not fully respond to nCRT. Molecular pathways underlying this variability are not fully understood, but it is believed that radioresistance among patients may contribute greatly.

Therapy resistance can be cancer-inherent, arising from preexisting cell subpopulations resistant to therapy, or acquired, when a cancer initially responds to treatment but relapses and progresses after a certain period. However, it is unclear whether rectal tumor radioresistance is inherent or acquired, or even a combination of both. Andel et al. established sensitive and radioresistant organoids derived from treatment-naïve RC patients and evaluated the evolution over time of cell subclones in response to radiotherapy [93]. Interestingly, irradiation of resistant organoids promoted subclonal persistence, and preexistent cell subpopulations with inherent radioresistance survived to therapy. Conversely, radiotherapy resistance occurrence in sensitive organoids befell because of non-dominant pre-irradiation subclone expansion which, after radiotherapy, became prevalent, so-called subclonal expansion. Moreover, radiosensitive cell clusters were eliminated, supporting the hypothesis that RC radioresistance is an inherent process caused by the expansion or persistence of preexisting cell subpopulations [93].

As previously mentioned, radiotherapy exerts its anti-tumor effect directly through DNA damage, more specifically by promoting double-strand DNA breaks (DSBs), or indirectly by water ionization, producing reactive oxygen species (ROS), which are molecularly unstable and induce cell damage [89, 90, 94]. However, despite its complexity, one of the major mechanisms underlying RC resistance is the enhanced ability of tumor cells to repair DNA damage. The non-homologous end joining (NHEJ) composes a primary pathway for DSBs repair, namely during the cell cycle G1 phase. Ku70/Ku80 heterodimer recognizes and binds to DSBs, recruiting DNA-dependent protein kinase catalytic subunit (DNA-PKcs), which together form the DNA-PK complex. Subsequently, if the DNA ends are incompatible, nucleases such as Artemis become active and process them, allowing the XRCC4-Ligase IV complex to catalyze their binding [95]. In a study carried out by Yu et al., involving 80 patients with LARC receiving neoadjuvant radiotherapy, poor or non-regression (Tumor regression grade (TRG) 3) exhibited PR-domain-containing proteins 15 (PRDM15) overexpression when compared to radioresponsive patients [96]. Interestingly, in vitro results showed that PRDM15 levels increased upon cell irradiation (8 Gy) and co-localized in high-DSBs regions, suggesting its recruitment to these regions. Moreover, it was noted that PRDM15 interacts directly with the DNA-PK complex, providing cells with a greater ability to repair DSBs, contributing to radioresistance. The PRDM15 role was further evaluated in cell lines and patient-derived xenograft models, and PRDM15 silencing compromising DSBs repair system enhanced radiotherapy effects [96]. Others have shown that irradiation increases EGFR internalization and nuclear localization, where it regulates the ku70/ku80 heterodimer expression in the radioresistant SW837 rectal cell line [97]. Besides, the authors noted that EGFR internalization and nuclear localization was Rab5C-dependent, a key regulator of the endocytic pathway. Hence, *RAB5C* silencing prevented EGFR internalization, increasing the cell susceptibility to radiotherapy [97]. Furthermore, given the pivotal role of the DNA-PK complex, Smithson et al. assessed the benefits of a DNA-PK complex inhibitor, Peposertib, in combination with nCRT [98]. Despite the benefits of Peposertib combination in vitro (8-fold DNA-PK expression decrease in combination with radiotherapy and 14-fold decrease with chemoradiation), in vivo results did not show significant improvements in pCR rates (18.2% for chemoradiotherapy + Peposertib vs. 12.5% for chemoradiotherapy alone) or tumor size, suggesting potential development of resistance mechanisms to treatment. To evaluate this, the authors found increased levels of phosphorylated ataxia-telangiectasia mutated (pATM) and KAP1 in cell lines exposed to chemoradiotherapy + Peposertib, proving the activation of alternative DSBs repair cascades [98].

**Fig. 4:**
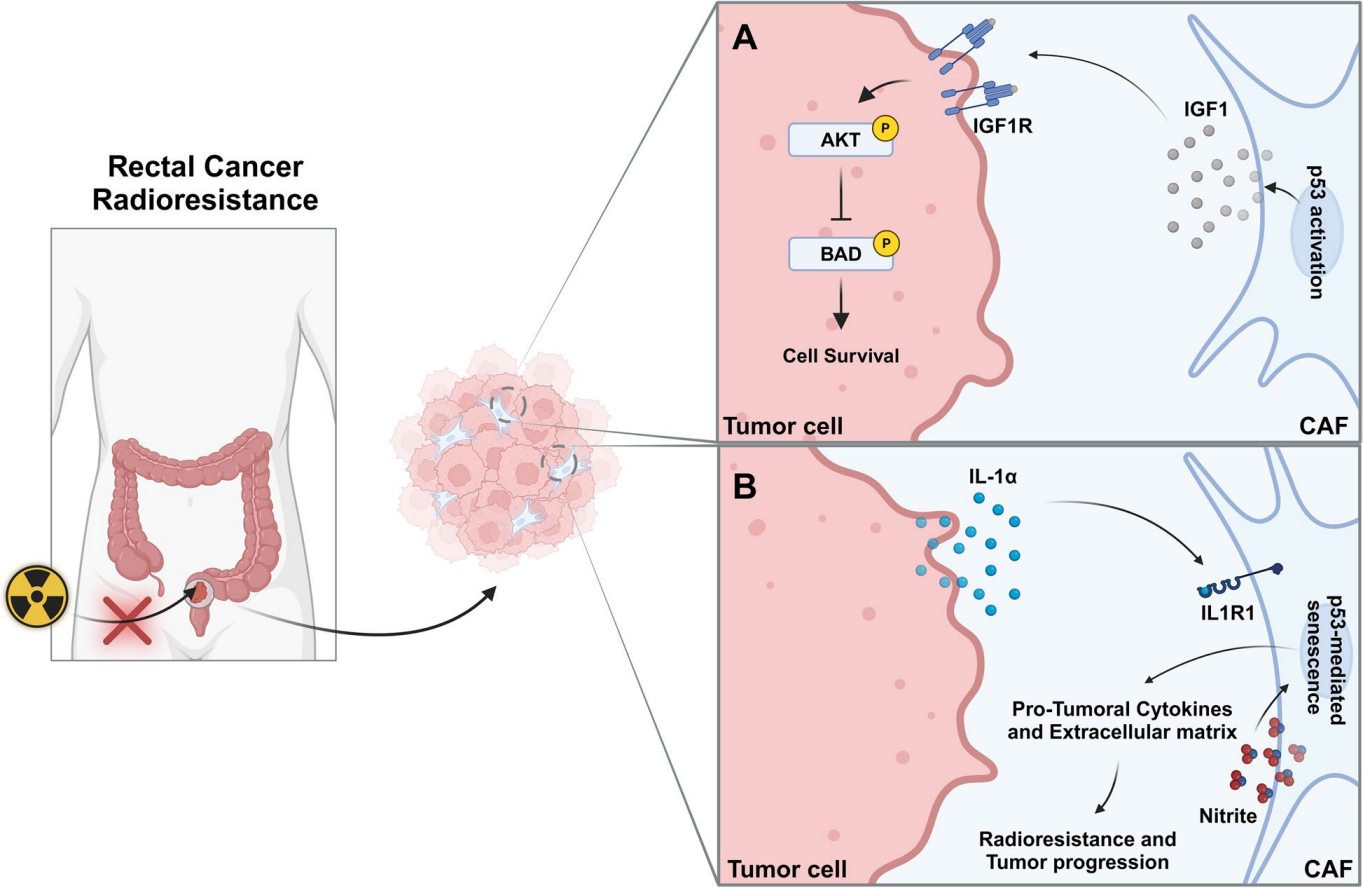
Tumor cell and cancer-associated fibroblasts crosstalk mediates radioresistance in rectal cancer.** A** p53 activation in response to radiotherapy-induced DNA damage leads to IGF1 expression and release by CAFs. IGF1-IGFR1 interaction triggers AKT/mTOR signaling, inhibiting the pro-apoptotic factor Bad and fostering cell survival and radioresistance in rectal cancer. **B** IL-1α release by tumor cells following irradiation triggers an inflammatory CAF polarization and nitrite release. Together, nitrite and radiotherapy-induced DNA damage drive CAFs senescence and cytokines and extracellular matrix constituents release, supporting tumor resistance and progression. IGF1, Insulin-like growth factor 1; CAF, Cancer-associated fibroblast

Differently, some studies pinpoint the important role of the TME, namely of cancer-associated fibroblasts (CAFs), in mediating RC radioresistance. Indeed, rectal tumors commonly exhibit a desmoplastic stroma with high levels of fibrosis and CAFs infiltration [99]. Pre-treatment biopsies from neoadjuvant therapy-resistant patients revealed enriched expression for CAFs, epithelial-mesenchymal transition and inflammatory markers [100]. To assess the influence of CAFs on radiotherapy response, Nicolas et al. established an orthotopic C57BL/6 mice model with organoids that, upon injection, exhibit CAFs-associated genes upregulation, as observed in RC patients [100]. Surprisingly, in vitro radioresponsive organoids shift to a radioresistant phenotype upon injection into mice and subsequent fractionated radiotherapy (5 × 2 Gy), highlighting a pivotal role of the tumor stroma in the development of radioresistance. Interestingly, exposure to the supernatant of these spheroids induced a pro-inflammatory phenotype in primary intestinal fibroblasts. This pro-inflammatory polarization was found to be interleukin 1α (IL-1α) signaling dependent, as inhibition with an IL-1 receptor (IL1R1) antagonist (anakinra) reversed it. Furthermore, to prove the critical role played by CAFs in acquired radioresistance, the authors evaluated the response of orthotopically transplanted organoids in *Col1a2*CreER^T2^*Il1r*^F/F^ knockout mice, characterized by *ILIR* silencing, particularly in CAFs. Strikingly, in the absence of IL1R1 signaling, sensitiveness to ionizing radiation was restored in organoids resulting in tumor regression. Additionally, radiotherapy activated a p53-dependent senescence program in pre-treated primary fibroblasts with organoid conditioned medium, as a consequence of excessive DNA damage caused by nitrite production by CAFs and radiation. Thus, radioresistance arises from an IL-1α-dependent polarization of CAFs leading to nitrite production which, combined with radiation-induced DNA damage triggers CAFs senescence, and subsequently the release of cytokines and of extracellular matrix constituents that support tumor progression (Fig. 4A) [100]. Similarly, poor radiotherapy responders exhibit lower levels of interleukin 1 receptor antagonist (IL-1ra). Consistently, human intestinal fibroblasts exposed to IL-1ra low pre-treatment polarize towards a pro-inflammatory phenotype, characterized by high nitrite production and SA-β-gal (senescence marker) staining, endorsing the IL-1 signaling pathway role in the neoadjuvant therapy response [100]. Considering these promising results, an ACO/ARO/AIO-21 phase I trial is currently undergoing to evaluate the benefits of anakinra combination with neoadjuvant capecitabine-based chemoradiotherapy for RC treatment [101]. The complex crosstalk between CAFs and RC cells has also been evaluated by Tommelein et al., where it was reported that irradiated CRC cells exhibited increased AKT activation and consequent inactivation of its downstream BAD (pro-apoptotic signal), when exposed to conditioned medium from irradiated fibroblasts (10 × 1.8 Gy/day) [99]. Besides, radiotherapy incited insulin-like growth factor 1 (IGF1) release by CAFs, who acts through a paracrine manner on the surrounding tumor cells, promoting IGF1 receptor (IGF1R) phosphorylation and AKT/mTOR signaling activation, culminating in the inhibition of the pro-apoptotic factor BAD and cell survival [99]. Although the exact mechanism underlying radiotherapy-induced IGF1 release by CAFs was not explored, the authors hypothesize that p53 activation in response to DNA damage upregulates p21, which prevents cyclin-dependent kinase 2 (CDK2) phosphorylation on nuclear transcription factor Y subunit alpha (NF-YA), driving *IGF1* expression (Fig. 4B) [99].

**Fig. 5:**
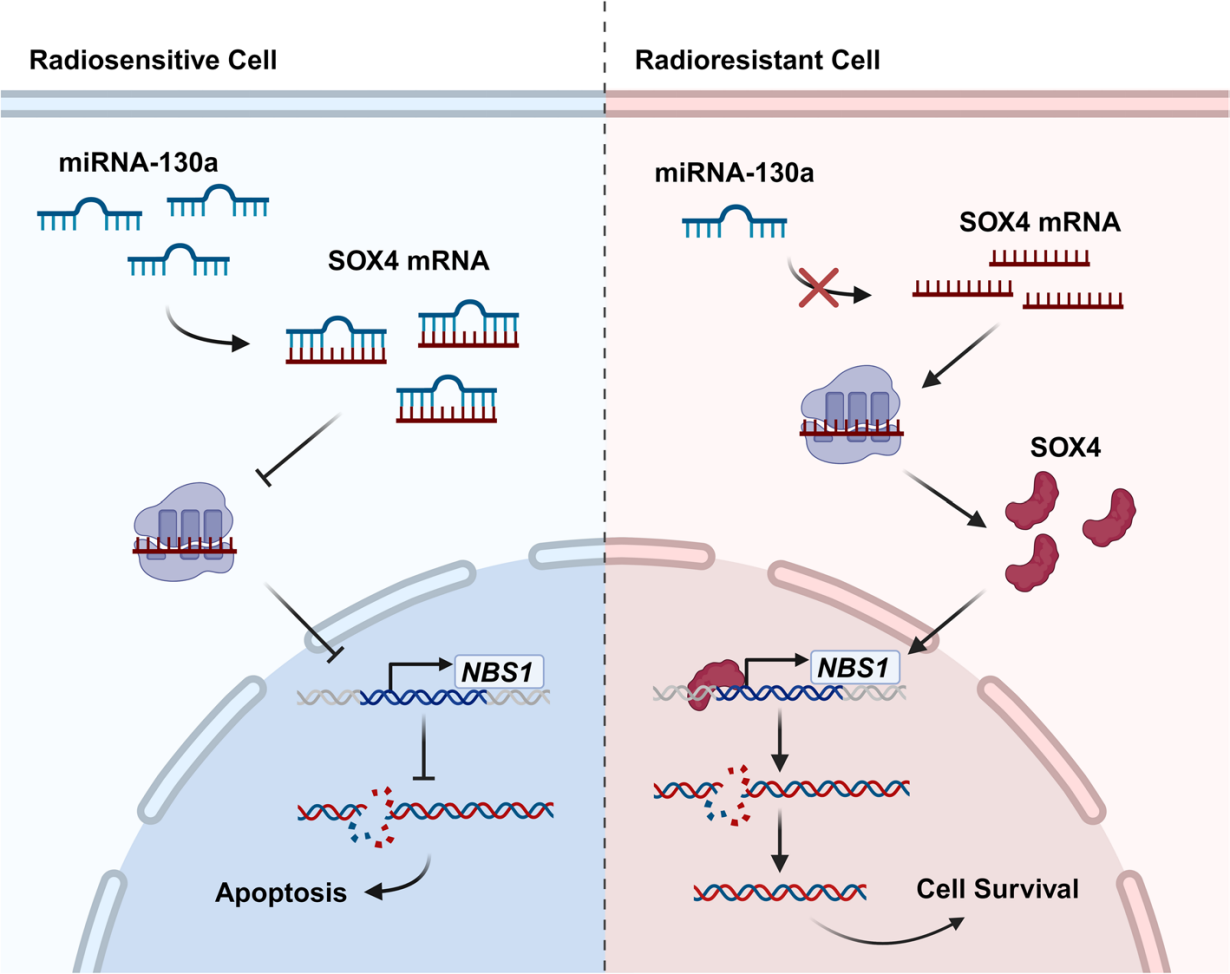
miRNA-130a modulates rectal cancer cell response to radiotherapy. High levels of miRNA-130a sensitize rectal tumor cell to radiotherapy by negative regulation on SOX4, downregulating NBS1, involved in DSBs repair, enabling tumor cells to repair radiation-induced DNA damage. DSBs, Double-strand DNA breaks; SOX4, SRY-Box transcription factor 4

Cancer stem cells constitute an important cell population within the TME, characterized by their self-renewal and differentiation potential [102, 103]. Furthermore, these cells are central in therapy resistance development in several types of cancer. In vitro studies revealed that cancer stem cells CD133^+^ were more radioresistant than CD133^−^ cells, and that the long non-coding RNA (lncRNA) DLGAP1-AS2 controls this [104]. LncRNA DLGAP1-AS2 demonstrated the potential to upregulate CD151 by transcription factor E2F1 modulation, leading to the AKT/mTOR/cyclin D1 cascade activation, fostering cell survival and proliferation [104]. Further, in vitro studies pointed out the miRNA-130a role in the radioresistance mediation in RC through negative regulation on SRY-Box Transcription Factor 4 (SOX4) mRNA [105]. Radiosensitive RC cells exhibit high levels of miRNA-130a, which by negative regulation on SOX4 downregulates NBS1, member of the MRN complex essential for DSBs recognition and activation of the protein kinase ATM, involved in DSBs repair, the most common type of DNA damage caused by ionizing radiation **(**Fig. 5**)**. Moreover, this was corroborated in a RC xenograft model, showing that the tumor suppressor miR-130a radiosensitizes RC cells by ATM suppression, compromising cells ability to fix DNA damage induced by radiation [105]. Relevantly, a study involving 40 patients with LARC supports the involvement of this miRNA on radioresistance, revealing low levels of miRNA-130a among non-responders patients, highlighting its potential as a predictive biomarker [106].

Fractionated ionizing radiation schemes allow the redistribution of the cancer cell cycle. It is known that the radiosensitivity of cells varies depending on their position in the cell cycle. Specifically, cells in the S phase are more resistant to radiation, while those in the G1, G2, and M phases are more sensitive [107]. By using fractionated doses, cells in the G2 and M phases are more effectively targeted to apoptosis during the first doses of radiation, while cells in the resistant S phase become more vulnerable to subsequent fractions of radiation by accumulation of DNA damage events [107]. Indeed, *TP53* mutations correlate with radioresistance predisposition in RC [108, 109]. The protein p53 orchestrates the cellular response to DNA damage via cell cycle arrest at G1 phase through its downstream target p21. However, its mutant form loses this capacity and cancer cells survive and proliferate. Immunohistochemical staining of wild type p53 is typically weak to moderate, but when mutated it is easier to detect due to its increased half-life [110]. Immunohistochemical analysis of 49 RC biopsies revealed that 83.3% of p53-negative and p21-positive tumors were radiosensitive, while 95.5% of p53-positive and p21-negative patients were radioresistant and had a higher risk of local recurrence, highlighting the association between p53 and p21 status and radiotherapy response [110]. Also, Chen, Zhou and colleagues showed that RIOK1, a protein kinase critical for cell cycle progression and ribosomal biogenesis, is up-regulated in paraffin-embedded rectal tumor tissue with increased TRG values [111]. The authors have further correlate clinical pathological data with RIOKI expression and stated that it significantly negatively associated with nCRT response and positively associated with tumor recurrence, with concomitantly decreased overall and disease-free survival (77.1% vs. 92.6%), (75.6% vs. 92.7%), respectively. Additionally, by silencing RIOK1, the authors showed in vitro and in vivo an enhanced sensitivity by decreasing cellular proliferation and colony formation capability, further showing that RIOK1 regulated p53 at post-transcriptional level by G3BP2 [111]. Therefore, radiotherapy resistance remains a major challenge in RC management and new therapeutic approaches to overcome nCRT drawbacks in RC are of utmost relevance.

## Nanomedicines in rectal cancer: bridging the gap in current therapies

Preoperative chemoradiotherapy is decisive for local control of advanced RC, and good responders can adopt a conservative sphincter-sparing strategy without compromising clinical outcomes. However, responses to nCRT vary significantly, and only up to 25% of patients can benefit from a watchful strategy, while the remaining experience minimal tumor regression or progression associated with poor prognosis. Therefore, novel approaches to boost the response rates to preoperative therapy in RC are of utmost importance.

NPs have gathered particular interest in the field of RC treatment over the past years. As outlined in this review, radioresistance plays a central role in the heterogeneity of responses to neoadjuvant and adjuvant therapy by patients with RC, by targeting not only mechanisms of DNA damage repair, but also the cell cycle and the TME [98, 100, 111]. NPs enhance drug pharmacokinetics by increasing the cell selectivity of drugs toward cancer cells, enhancing the intracellular drug accumulation of chemotherapeutics and radiosensitizers. Hence, nanocarriers have been extensively investigated to improve radiotherapy outcomes [112, 113]. Table 1 summarizes all nanosystems, specifically those designed for RC treatment.

**Table 1:** Novel nanomedicines proposed to improve neoadjuvant chemoradiotherapy response in rectal cancer

NP composition	Cargo	NP intrinsic abilities	Therapeutic Improvements	Reference
Cyclodextrin-based NP	Camptothecin	-	Counters HIF-1α overexpression induced by radiotherapy. Enhance DNA damage in combination with radiotherapy. Delay tumor progression in combination with nCRT vs. nCRT alone. pCR of 33% in a phase II trial.	[13, 114]
Hafnium oxide NP	-	ROS generation.	Tumor retention for more than two weeks. Improves disease control in combination with nCRT.	[115, 116]
Dendritic mesoporous silica nanocarrier with platinum-based NPs	cGAMP (STING agonist)	Counteract tumor hypoxia by its catalase-like catalytic activity. ROS generation.	Increase DSBs levels and apoptosis rate in combination with radiotherapy vs. radiotherapy alone. Fosters an anti-tumoral TME. In vivo combination with radiotherapy and anti-PD1 achieved a complete tumor regression in all mice. Ability to promote an immune response in metastasis.	[12]
Pegylated FePt NP functionalized with cRGDfk peptide	Doxorubicin	Inhibit replication forks slow down by its catalase-like activity. ROS generation.	Tumor targeting. Controlled drug release under acidic conditions. Ability to induce cell death by replication catastrophe. Counteract tumor hypoxia via HIF-1α downregulation. NPs combination with radiotherapy improves disease control compared to radiotherapy alone.	[117]
Heterojunction of BiVO_4_ photocatalyst with ZnIn_2_S_4_ nanosheets coated with poly-norepinephrine and macrophage membranes	-	Lactic acid rescue from the TME. ROS and carbon monoxide generation. Glutathione oxidation.	Tumor targeting. Increase ROS levels and apoptosis rate. Fosters an anti-tumoral TME by reducing lactic acid levels. Remarkable tumor regression in combination with optical fibers.	[118]
Pegylated DPPC and cholesterol liposome	Metformin and 2-DG	-	Increase apoptosis rate via eIF2α and ATF4 downregulation and higher mitochondrial permeabilization. Superior disease control in combination with radiotherapy compared to radiotherapy alone. Increases immune infiltration and reverses T cell exhaustion upon radiotherapy.	[119]
Pegylated PLGA NPs functionalized with TPP	Verteporfin and ultrasmall gold NPs	ROS generation.	Tumor uptake and mitochondria targeting. Enhance radiotherapy-induced apoptosis via BAX, BAD, and caspase-3 upregulation. Better tumor control and improves survival rate in combination with radiotherapy.	[120]
PLGA/DPPC hybrid NP	siPOLR2A and metformin bicarbonate	Endosomal membrane burst through carbon dioxide release.	Improves tumor retention. Endo/lysosomal escape and siPOLR2A deliver into the cytoplasm. Increases siRNA stability and efficacy, avoiding RNase degradation. Survival improvement with a 66% rate of complete tumor regression in mice with POLR2A hemizygous deletion.	[121]
Pegylated ZIF-8 NP functionalized with iRGD peptide	miRNA-181a and MnO_2_ NPs	ROS generation. Counters tumor hypoxia by its catalase activity.	Tumor targeting and uptake. Enhances miRNA stability, avoiding RNase degradation. Better tumor growth control in combination with radiotherapy compared to radiotherapy alone. Boost an antitumoral TME.	[122]
Pegylated albumin NP functionalized with B5 ligand	5-Fluorouracil	-	Tumor targeting. NPs combination with radiotherapy improves tumor progression control compared to free drug + radiotherapy.	[123]
Pegylated silicasome consisting of mesoporous silica NPs coated with a lipid bilayer (DSPC/ cholesterol/DSPE)	Irinotecan	-	In combination with radiotherapy, NPs enhanced its effects, increasing ROS and apoptosis levels. Irinotecan pharmacokinetics improvement, especially tumor accumulation (795-fold increase vs. free drug). Improved tumor growth control in combination with radiotherapy and anti-PD1 therapy, fostering an anti-tumoral TME.	[124]
Tricaprin SLN incorporated into a thermosensitive poloxamer solution	Irinotecan	-	Prolonged irinotecan half-life time and slower clearance. Increased tumor retention and better tumor progression control.	[125]

CRLX10, a cyclodextrin-based NP conjugated with camptothecin, developed by Cheng J. et al., has demonstrated its ability to increase DSBs levels in combination with radiotherapy [13, 126]. In agreement, CRLX10 addition to chemoradiotherapy in a RC xenograft mouse model improved tumor control growth [13]. Considering these results, CRLX10 combination with standard nCRT was evaluated in a phase II single-arm trial (Registration NCT02010567), showing a pCR rate of 33% for patients undergoing weekly maximum tolerated doses, without increased toxicity [114].

High atomic number (High-Z) materials such as platinum, gold, hafnium and selenium, have been subject of extensive research for NPs synthesis in RC due to their intrinsic radiosensitizing properties. These biomaterials exhibit a high capacity to absorb X radiation, increasing its deposition within the tumor. After internalization by tumor cells, irradiation triggers electrons release from these NPs that, in turn, hydrolyze water molecules generating ROS that cause DSBs in the DNA. NBTXR3, a hafnium oxide NP without cargo in phase Ib/II trial (Registration NCT02465593), was intratumorally injected to assess its radioenhancer potential, in combination with nCRT for LARC treatment [115]. Results proved the combination ability to completely control disease, with no evidence of tumor progression among the 31 LARC patients and a pCR rate of 20% [116].

**Fig. 6:**
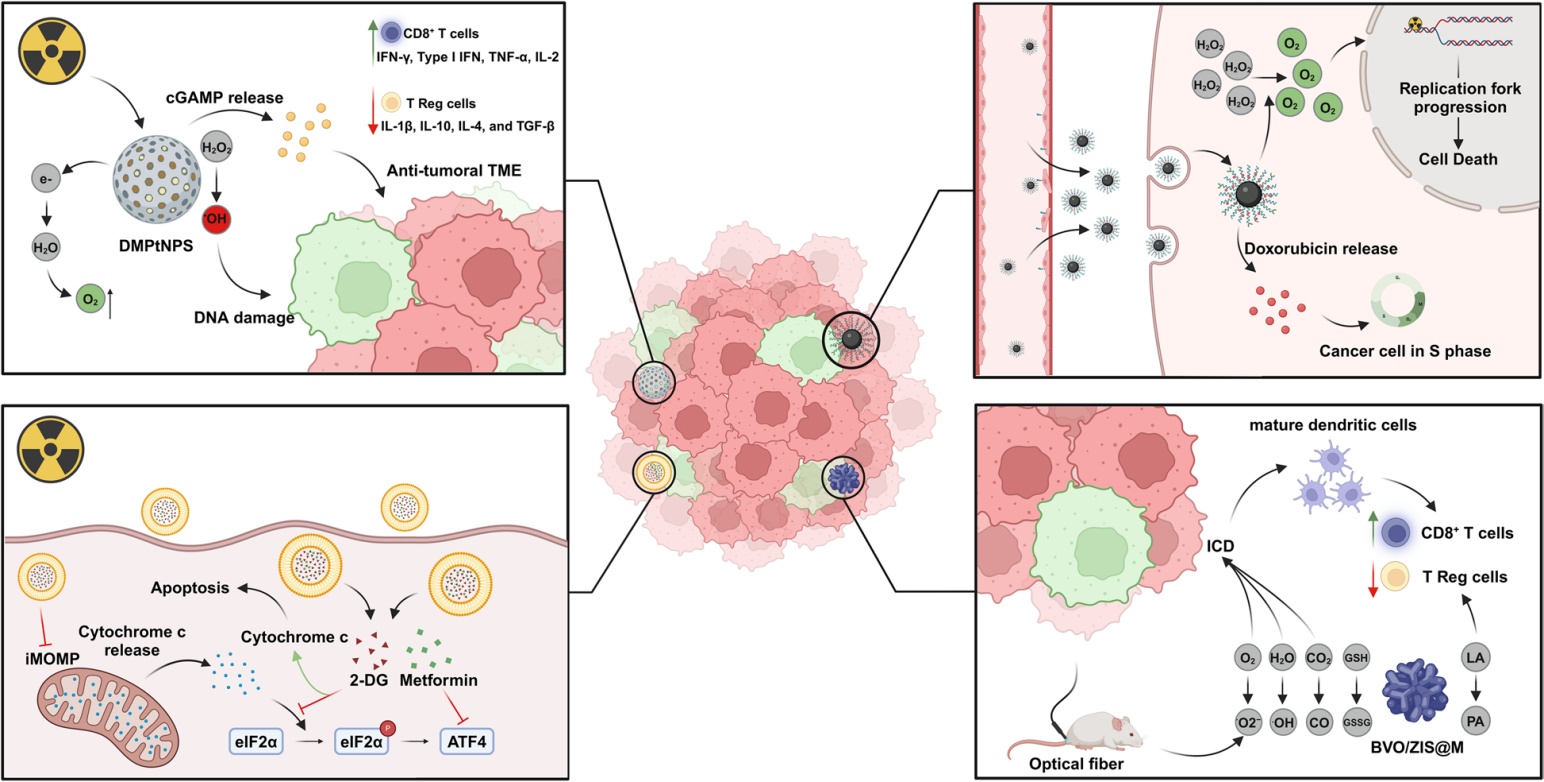
Schematic illustration of different nanomedicine applications in rectal cancer. Nanoparticles harbor intrinsic characteristics capable of radiosensitizing rectal tumors for radiotherapy while acting simultaneously as chemotherapeutic drugs carriers. ATF4, activating transcription factor 4; BVO/ZIS@M, quatrefoil-shaped heterojunction catalyst coated with poly-norepinephrine and macrophage membranes; DMPtNPS, dendritic mesoporous silica nanocarrier with platinum-based nanoparticles; GSH, glutathione; GSSC, oxidized glutathione; eIF2α, eukaryotic initiation factor 2α; ICD, immunogenic cell death; iMOMP, incomplete mitochondrial outer membrane permeabilization; LA, lactic acid; TME, tumor microenvironment; T reg – Regulatory T cell

Rectal tumors are typically hypoxic, with oxygen levels around 1.8%, less than half of normoxia levels of about 3.8% [127]. Hypoxia contributes to tumor radioresistance in RC by hypoxia-inducible factor 1-alpha (HIF-1α) upregulation in response to low oxygen levels, promoting cell survival and tumor progression [128]. To address the hypoxic TME in RC, Wang et al. developed a dendritic mesoporous silica nanocarrier with platinum-based nanoparticles (DMPtNPS) with inherent catalase-like activity to catalyze hydrogen peroxide (H_2_O_2_) into oxygen (O_2_) (Fig. 6) [12]. Compared to radiotherapy, the combination of DMPtNPS and radiotherapy significantly reduced CT26 and HCT116 cells viability from 59.9% and 57.5 to 25.7% and 17.4%, respectively. This effect resulted in superior intracellular ROS production, leading to higher levels of phosphorylated H2AX (γ-H2AX), a DSBs marker, increasing considerably cell apoptosis rate. Moreover, DMPtNPS and radiotherapy combination significantly enhanced tumor immunogenicity by increased expression of immunogenic cell death (ICD) biomarkers, such as calreticulin, also known as eat me signal, ATP and HMGB1. Consequently, dendritic cells preferentially matured into a type 1 anti-tumor phenotype (mDC1) fostering helper T cells differentiation into Th1 cells, an anti-tumor subset of T cells, through interleukin-12 and tumor necrosis factor-alpha (TNF-α) release. Also, tolerance-inducing T cell mediators, such IL-10 and TGF-β, were downregulated [12]. Unexpectedly, while the nanosystem revealed its ability to reduce tumor hypoxia in vivo and increased DNA damage, in combination with radiotherapy, sustained tumor growth inhibition was not achieved [12]. Cyclic GMP-AMP synthase (cGAS)-stimulator of interferon genes (STING) pathway is a key mediator of anti-tumor immunity. cGAS is responsible for recognizing cytosolic double-stranded DNA (dsDNA) and release 2′3′ cyclic GMP-AMP (cGAMP) that binds to STING, located in the endoplasmic reticulum membrane, promoting its translocation to Golgi, where it recruits and activates TANK-binding kinase 1. This in turn, lead to STING and interferon regulatory factor 3 phosphorylation, which dimerizes and translocates into the nucleus, inducing the transcription of pro-inflammatory genes [129]. Despite cGAS-STING cascade activation, programmed death-ligand 1 (PD-L1) downstream was also upregulated in tumor cells following DMPtNPS and radiotherapy combination. In addition, these tumors exhibited increased infiltration of regulatory T (T reg) cells and immunosuppressive cytokines upregulation, such IL-1β, IL-10, IL-4, and TGF-β [12]. To address this, the STING agonist, cGAMP, was encapsulated into DMPtNPS (DMPtNPS@cGAMP) in a CT26 tumor-bearing mouse model, which resulted in improved control tumor growth in combination with radiotherapy, with a sustained complete response of 30%. Remarkably, the authors noted that anti-PD1 therapy addition to the DMPtNPS@cGAMP and radiotherapy combination resulted in sustained complete responses for 60 days in 100% of primary tumors and in 50% of distant tumors in a bilateral tumor mouse model. The robust immune response against the primary tumor was extended to the secondary tumor, through a so-called abscopal effect. Importantly, no hepatic or renal disturbances were detected, either by histology or biochemical analysis [12].

Liao, Yang et al. established the development of an αvβ3 target pegylated catalytic FePt NP carrying doxorubicin, leveraging platinum inherent catalase-like activity, preventing the use of H_2_O_2_ as a mediator for Peroxiredoxin 2-induced replication forks slow down [117]. This disrupts DNA damage repair, inducing replication catastrophe and cell death (Fig. 6). In vivo results proved the system efficacy as an amplifier of radiotherapy-induced replicative stress, leading to increased DNA damage and reduced tumor hypoxic via HIF-1α downregulation, culminating in tumor volume reduction [117]. Recently, Li et al. developed a catalytic therapy employing a quatrefoil-shaped heterojunction catalyst coated with poly-norepinephrine and macrophage membranes (BVO/ZIS@M) capable of using lactic acid as an electron donor [118]. Furthermore, this platform exhibits an intrinsic ability to oxidize glutathione and increase ROS levels within the tumor, namely superoxide (O_2_^−^) and hydroxyl radical (·OH), as well as carbon monoxide (CO) after optical fiber intervention, causing DNA or mitochondria membrane damage, and drastically increasing the percentage of tumor cells undergoing apoptosis (Fig. 6). Moreover, the reduction of lactic acid levels within the TME resulted in fewer T regs and increased CD8^+^ T cells. To overcome one of the main problems in photocatalytic therapy related to limited light penetration depth, the authors used optical fibers inserted into the mice anus [118]. In vivo biodistribution studies demonstrated tumor accumulation after 6 h of oral administration, persisting for a least 12 h. Hence, light irradiation therapy was given 7 h after oral system administration of BVO/ZIS@M. The combination-treated therapy group exhibited remarkable tumor suppressive ability, attributed to high levels of apoptosis and a robust antitumor immune response [118].

The Eukaryotic initiation factor 2α (eIF2α) and its downstream activating transcription factor 4 (ATF4) overexpression is commonly observed in CRC patients with poor radiotherapy response and is linked to incomplete mitochondrial membrane permeabilization, promoting cell survival [119, 130]. To counteract this, Li et al. developed a mitochondria-modulating liposome co-encapsulating metformin and 2-DG, capable of reversing p-eIF2α and ATF4 increased levels, respectively [119]. Additionally, by eIF2α and ATF4 downregulation, treatment combination of radiotherapy and liposomes (2 Gy + Liposomes + 8 Gy) enhanced the apoptosis rate of CT26 cells, given the increased mitochondrial membrane permeabilization, allowing the accumulation in the cytoplasm of the pro-apoptotic signal cytochrome C (Fig. 6). In a CT26 tumor-bearing mice model, liposomes significantly improved radiotherapy effects, extending survival from 24 days to over 40 days in the combination-treated group, with no evidence of associated toxicity [119]. This was followed by a shift from an immunosuppressive to a tumor-reactive TME, with increased CD3^+^ T cell infiltration and reduced Tim-3^+^ PD-1^+^ T cells and PD-L1 levels, highlighting the system ability to reverse T cell exhaustion following radiation. This ability of the nanosystem was also witnessed at the metastatic nich, in a bilateral model [119].

Mitochondria targeting was also a strategy used by Deng et al., who developed pegylated polymeric NPs functionalized with triphenylphosphonium (TPP) to target the inner mitochondrial membrane, exploiting the reduced mitochondrial membrane potential of tumor cells [120]. Poly lactic-co-glycolic acid (PLGA) NPs carrying the photosensitizer verteporfin and ultrasmall gold NPs showed the ability to generate ROS, namely singlet oxygen (^1^O_2_) upon low doses of X-radiation. NP functionalization enhanced cellular uptake and mitochondrial localization, which in combination with radiotherapy (4 Gy single dose) led to increased ^1^O_2_ production, due to the mitochondria oxygen-rich environment. Consequently, elevated ^1^O_2_ levels induced mitochondria damage dipping mitochondrial membrane potential, and impairing ATP synthesis. Besides that, mitochondrial pro-apoptotic proteins BAX, BAD, and caspase-3 were upregulated while the anti-apoptotic Bcl-2 was downregulated upon NPs and radiotherapy combination, emphasizing the nanocarrier ability to potentiate the apoptotic effects of radiotherapy [120]. In vivo studies revealed the therapeutic combination efficacy to reduce tumor size and prolong survival rates [120].

*RNA Polymerase II Subunit A* (*POLR2A*) is located on chromosome 17p13.1 close to *TP53*, and in 67% of RC cases is hemizygously co-deleted with *TP53* [121]. However, one allele of the *POLR2A* gene is sufficient to sustain cancer cells survival [131]. To overcome this, a siPOLR2A was encapsulated into a polymer/lipid hybrid NP consisting of PLGA and dipalmitoylphosphatidylcholine (DPPC) [121]. RNA-interference-based therapies for cancer treatment suffer from several challenges, including low bioavailability due to siRNA instability in the blood plasma and limited cell selectivity. Besides that, evading lyso- and endosomes, where RNases are found, is critical to siRNA therapy success. Therefore, Xu et al. devised a strategy based on the metformin capacity to capture carbon dioxide via its biguanide group at neutral pH and release it at acidic pH, disrupting the endosome membrane [121]. Carbon dioxide release was confirmed by physicochemical alterations in the NPs upon acidic pH exposure, namely increased hydrodynamic diameter. Cell viability assays confirmed the nanoplatform efficacy to prevent endosomal entrapment and facilitate cytosol delivery of siPOLR2A in SW837 RC cells, reducing cell viability, particularly those with hemizygous deletion of *POLR2A* [121]. In vivo assays demonstrated the NPs capacity to retain siPOLR2A within tumor derived from isogenic POLR2A-deficient cells for over 24 h. Furthermore, NPs demonstrated remarkable tumor growth suppression. Notable, all mice were alive after 56 days with a 66% complete tumor regression rate [121]. To deliver miR-181a, known to enhance radiosensitivity, Hao et al. engineered an αvβ3 integrin target pegylated nanoplatform consisting of zinc ions conjugated to the nitrogens of 2- methylimidazole carrying miR-181a and manganese dioxide NPs [122]. In combination with radiotherapy, those NPs increased in vivo ROS generation and DNA damage, highlighting their radiosensitizer ability. Furthermore, NPs exhibited intrinsic catalase-like activity, mitigating radiotherapy-induced hypoxia, converting H_2_O_2_ to O_2_ and subsequently downregulating HIF-1α. Lastly, the therapeutic combination boosted the antitumoral immune response, increasing CD4^+^ CD44^high^ and CD8^+^ CD44^high^ T cell tumor infiltration [122].

5-FU chemotherapeutic regimens are the mainstream option for neoadjuvant therapy in RC. Lee et al. developed a pegylated albumin-based NP for 5-FU delivery [123]. NPs were functionalized with a B5 ligand (Peptide Sequence: TPSFSKI) to target LRP-1, a protein identified by the same authors as a marker of radioresistance. In vivo results have demonstrated the benefits of 5-FU encapsulation and NP functionalization to significantly improve radiotherapy efficacy [123]. Long-term results from the phase III PRODIGE 23 trial demonstrated that irinotecan, used as induction chemotherapy (mFOLFIRINOX scheme) followed by nCRT, surgery and adjuvant chemotherapy improved the 7-year overall survival (81.9% vs. 76.1%), and disease-free survival (67.6% vs. 62.5%) compared to the gold standard therapy [132]. However, irinotecan introduction into RC chemotherapeutic regimens faces several challenges, such as side effects like diarrhea and neutropenia, and fast drug clearance [133]. To address those drawbacks, irinotecan was encapsulated into a pegylated silicasome consisting of mesoporous silica NPs coated with a lipid bilayer, and its synergistic potential in combination with radiotherapy was evaluated by Wang et al. [124]. NPs strengthened ionizing radiation effects, increasing ROS and apoptosis levels compared to radiotherapy alone. Furthermore, increased cytosol dsDNA levels were observed after NPs and radiotherapy combination, thereby enhancing cGAS-STING signaling pathways activation [124]. In vivo pharmacokinetic analysis revealed a dramatic increase in tumor irinotecan concentration upon encapsulation, reaching levels 795-fold higher than free drug after 48 h, allowing a significant improvement in tumor growth control [124]. Din et al. encapsulated irinotecan into solid lipid nanoparticles with further incorporation into a thermosensitive solution that jellifies at rectum temperature [125]. NPs improved irinotecan pharmacokinetics, namely prolonged half-life time and reduced clearance. Moreover, NPs incorporation into the thermosensitive system enhanced in vivo tumor growth control, evidenced by PARP and caspase-3 overexpression and decreased ki-67 levels [125].

## Conclusions and future perspectives

RC has a different molecular landscape from CC, especially from those arising in the proximal colon. Although tumors from the large bowel are grouped as CRC, they develop through distinct molecular pathways and the tumor anatomical site influences its carcinogenesis. A deep understanding of those differences is crucial to develop targeted therapies and optimize treatment strategies for improved patient outcomes. Therapy resistance, mainly radioresistance, is a major therapeutic hurdle in RC associated with poor prognosis. Dysregulated molecular pathways associated with DNA damage repair, cell cycle distribution and the influence of the TME pave the way for the intrinsic or acquired radioresistance mechanisms. Identifying treatment predictive markers is paramount to minimize unnecessary therapy exposure to patients. Whilst the underlying molecular mechanisms of radioresistance in RC are diverse, a common thread appears to be the presence of cells with enhanced capacity to repair DSBs induced by ionizing radiation. Multifunctional nanomedicines offer a way to improve radiotherapy response by enhanced DNA damage, both by inherent characteristics, namely those consisting of high-Z metals, or by encapsulation of radiosensitizing drugs, such as 5-FU and irinotecan. Intriguingly, despite 5-FU regimens being the gold standard for nCRT in RC, only one study by Lee et al. has explored the benefits of its encapsulation into NPs [123]. Intravenously 5-FU exhibits short half-life and undergoes fast hepatic metabolism by dihydropyrimidine dehydrogenase into inactive metabolites, hampering 5-FU tumor accumulation [134, 135]. Consequently, 5-FU requires high doses associated with significant toxicity. Therefore, nano-enabled strategies are required to increase tumor accumulation. However, despite promising results, several challenges hinder nanomedicine translation ability to the clinical setting, such as the lack of nanoparticles long-term effects on the human body and the need to implement good manufacturing practices [136]. Furthermore, despite significant efforts to uncover mechanisms of radioresistance and the potential of nanosystems to overcome them, most in vitro and in vivo models fail to recapitulate the human RC panorama. To the best of our knowledge, no murine RC cell line is currently available, and the development of an orthotopic murine model for RC remains an ongoing challenge. Consequently, most of the studies are performed with CC models. As we described, tumors arising throughout the large intestine exhibit a continuum spectrum of genetic features, such MSS, *TP53* and *BRAF* mutations prevalence. Therefore, rectal tumors have distinct traits compared to colon tumors, especially those from the proximal colon. Recently, significant efforts have been made to develop RC models [137–139]. Kim et al. established an orthotopic mouse model for RC by engrafting genetically modified *APC*^*-/-*^, *KRAS*^*G12D/+*^ and *TP53*^*-/-*^ organoids, derived from mouse rectal tissue [137]. Tumors exhibited the ability to evolve to an invasive stage and mirrored M2 macrophage infiltration after irradiation, commonly observed in the clinical setting [137, 140]. Notably, this model overcomes limitations of others, such dextran sulfate sodium-induced colitis, which alters the TME, or intrarectal injection of tumor cells, which bypasses RC natural progression from the mucosa [137]. Furthermore, patient-derived organoids (PDOs) have proved their ability to accurately preserve the genetic background of their paired primary tumors and have proven to be powerful tools to predict patient responses to treatment with high sensitivity and accuracy [138, 139]. Ganesh et al. evaluated the correlation between the sensitivity of PDOs to 5-FU or the FOLFOX regimen and the progression-free survival of patients, finding a strong correlation (Spearman *r* = 0.86) [138]. Furthermore, PDOs established by Yao et al. highly correlated with actual patients response to therapy, with 88.20% AUC, 84.43% accuracy, 78.01% sensitivity and 91.97% specificity [139].

Additionally, work from our own group and collaborators is focused on the development and characterization of two low-cost, easy-to-use, and high-throughput RC models for radiobiology studies. These preclinical models provide a reliable platform for studying radioresistance and pave the way for the development and accelerated evaluation of novel, more effective therapies to overcome radiotherapy resistance in RC. Recently, dose escalation has garnered significant attention for its potential to enhance tumor regression and oncological outcomes [141, 142]. Additionally, the multicenter phase III OPERA trial demonstrated that 90 Gy/ 3 fractions boost, delivered by contact X-ray brachytherapy 1 or 2 weeks before neoadjuvant chemoradiotherapy, resulted in a remarkable 3-year organ preservation rate of 97% in cT2-cT3 RC patients with tumors smaller than 3 cm of diameter [142]. However, it remains unclear the impact of dose escalation on the molecular mechanisms underlying radioresistance in RC and the potential to shift semi-radioresistant or radioresistant tumors towards sensitive ones.

To conclude, future research should prioritize the development of more robust models that accurately recapitulate the genetic panorama and TME found in rectal tumors, which will be critical to uncover and confirm the underlying mechanisms of radiotherapy resistance and to evaluate the therapeutic potential of NPs as an emerging strategy to overcome radioresistance in RC.

## Data Availability

No datasets were generated or analysed during the current study.

## Abbreviations

5-FU: 5-Fluorouracil
APC: Adenomatous Polyposis Coli
ATF4: Activating transcription factor 4
ATM: Ataxia–telangiectasia mutated
CAF: Cancer–associated fibroblast
CC: Colon cancer
CDK2: Cyclin–dependent kinase 2
cGAMP: 2′3′ cyclic GMP–AMP
cGAS: Cyclic GMP–AMP synthase
CIN: Chromosomal instability
CIMP: CpG island methylator phenotype
CMS: Consensus Molecular Subtype
CRC: Colorectal cancer
CTNNB1: Catenin Beta 1
dMMR: Mismatch repair deficiency
DMPtNPS: Dendritic mesoporous silica nanocarrier with platinum–based nanoparticles
DNA: PKcs–DNA–dependent protein kinase catalytic subunit
DPPC: Dipalmitoylphosphatidylcholine
DSB: Double–strand DNA break
dsDNA: Double–stranded DNA
EGFR: Epidermal growth factor receptor
eIF2α: Eukaryotic initiation factor 2α
EPR: Enhanced permeability and retention
ESMO: European Society for Medical Oncology
HIF-1α: Hypoxia-inducible factor 1-alpha
High-Z: High atomic number
ICD: Immunogenic cell death
IGF1: Insulin–like growth factor 1
IGF1R: Insulin–like growth factor 1 receptor
IL-1α: Interleukin 1α
IL1R1: Interleukin 1 receptor, type 1
IL-1ra: Interleukin 1 receptor antagonist
iMOMP: Incomplete mitochondrial outer membrane permeabilization
LARC: Locally advanced rectal cancer
LCPRT: Long–course preoperative radiotherapy
lncRNA: long non–coding RNA
NCCN: National Comprehensive Cancer Network
nCRT: Neoadjuvant chemoradiotherapy
NF-YA: Nuclear transcription factor Y subunit alpha
NHEJ: Non–homologous end joining
NP: Nanoparticle
MAPK: Mitogen–Activated Protein Kinase
MLH1: MutL Homolog 1
MMR: Mismatch repair
MSH2: MutS Homolog 2
MSI: Microsatellite Instability
MSI-H: MSI-high
MSS: Microsatellite stability
pCR: Pathological complete response
PD-1: Programmed cell death-1
PD-L1: Programmed death–ligand 1
PDOs: Patient–derived organoids
PI3K: Phosphoinositide 3–Kinase
PLGA: Poly lactic–co–glycolic acid
POLR2A: RNA Polymerase II Subunit A
PRDM15: PR–domain–containing proteins 15
RC: Rectal cancer
ROS: Reactive oxygen species
SCPRT: Short–course preoperative radiotherapy
SOX4: SRY–Box Transcription Factor 4
STING: Stimulator of interferon genes
TME: Tumor microenvironment
TNF-α: Tumor necrosis factor-alpha
TNT: Total neoadjuvant therapy
TP53: Tumor protein p53
TPP: Triphenylphosphonium
T reg: Regulatory T cell
γ-H2AX: phosphorylated H2AX
